# Between 2000 and 2020, Reimbursement for Orthopaedic Foot and Ankle Surgery Decreased by 30%

**DOI:** 10.1016/j.asmr.2021.11.016

**Published:** 2021-12-24

**Authors:** Jordan R. Pollock, M. Lane Moore, Jack M. Haglin, Matthew P. LeBlanc, Christian S. Rosenow, Justin L. Makovicka, David G. Deckey, Jeffrey D. Hassebrock, Joshua S. Bingham, Karan A. Patel

**Affiliations:** a Mayo Clinic Alix School of Medicine, Scottsdale, Arizona, U.S.A.; b Department of Orthopedic Surgery, Mayo Clinic, Phoenix, Arizona, U.S.A.; c Texas Tech University, Lubbock, Texas, U.S.A.

## Abstract

**Purpose:**

To examine and analyze Medicare reimbursement rates from 2000 to 2020 for orthopaedic foot and ankle procedures.

**Methods:**

The 20 most used orthopaedic foot and ankle surgical procedures were gathered from the Centers for Medicare & Medicaid Services website using the Medicare Provider Utilization and Payment Data Public Use File 2017. The reimbursement data for each code were gathered from The Physician Fee Schedule Look-Up Tool from Centers for Medicare & Medicaid Services. The reimbursement values were adjusted for inflation to 2020 U.S. dollars using the consumer price index.

**Results:**

The average inflation-adjusted reimbursement for included procedures decreased by 30% from 2000 to 2020. The greatest mean decreases were observed for “correction of hallux valgus” (–47%) and “partial excision of foot bone” (–41%). The procedures with the smallest mean decreases were observed in “treatment of “Amputation of toe” (–19%) and “closed treatment of metatarsal fracture” (–7%).

**Conclusions:**

From 2000 to 2020, Inflation-adjusted Medicare reimbursement for foot and ankle surgery decreased by 30%.

**Level of Evidence:**

IV; economic analysis.

Health care billing in the United States is a complex, multipayer system, with Medicare being the largest payer.^1^ In the current system, federal and private payers reimburse physician services based on physician billing. One of the primary physician billing code sets is the Current Procedural Terminology (CPT), for which each code is assigned a reimbursement value that is based on several factors such as work, practice expense, and malpractice expense. These factors are quantified using relative value units and converted into a specific payment rate for each CPT code.^2^

Within the literature, it has been reported that the prevalence of ankle procedures, such as ankle arthroscopy, has steadily increased within the United States.^3^ This growth has even outpaced that of shoulder, knee, and elbow arthroscopy.^3^ Despite this increase, few research investigations exist that analyze the trends in reimbursement for these types of procedures. Medicare reimbursement trends have been quantified in specialties such as emergency medicine, orthopaedic surgery subspecialties, general surgery, oncology, and neurosurgery.^4^, ^5^, ^6^, ^7^, ^8^, ^9^, ^10^, ^11^, ^12^ The purposes of this study were to examine and analyze Medicare reimbursement rates from 2000 to 2020 for orthopaedic foot and ankle procedures. We hypothesized that Medicare reimbursement, when adjusted for inflation, would have decreased substantially over the past 2 decades for all commonly performed procedures by foot and ankle surgeons.

## Methods

The 20 most used orthopaedic foot and ankle surgical procedures (CPT codes 27600-28899) were gathered from the Centers for Medicare & Medicaid Services (CMS) website using the Medicare Provider Utilization and Payment Data Public Use Files for Calendar Year 2017 visualization tool.^13^ This tool contains data published by CMS relating to procedure use. Financial data for each of the included CPT codes were accessed using The Physician Fee Schedule Look-Up Tool.^14^ The extracted pricing information for all geographic Medicare Administrative Contractors across the country from 2000 to 2020 were averaged across all Medicare Administrative Contractors to obtain a national average for each service. The unadjusted percentage change was calculated for each procedure and all procedures were averaged to obtain an overall mean percentage change for the top 20 CPT codes. To compare the rate of change to reimbursement with the rate of inflation, the average unadjusted reimbursement value was taken and compared to the percentage change in Consumer Price Index (CPI) using a 2-tailed *t*-test comparison of means with alpha of 0.05 set as significant.

The most current CPI data (January 2020) was obtained from the U.S. Department of Labor, Bureau of Labor Statistics.^15^ This CPI was then used to adjust reimbursement values for inflation to 2020 U.S. dollars. The average annual, total percentage change, and adjusted R-squared values were calculated after adjusting for inflation for each procedure, and on average across all procedures. The compound annual growth rate (CAGR) also was calculated, an economic investments measure for change over time, with the adjusted data in 2020 dollars using the following formula:

$$\text{CAGR}={\left(2020\;\text{Value}/2000\;\text{Value}\right)}^{1/\left(2020-2000\right)}-1$$

All statistics and calculations were performed using Microsoft Excel for Office 365 (Microsoft Corp, Redmond, WA). Institutional review board approval was not required for this study as only publicly available data was used in our analysis.

## Results

From 2000 to 2020, the average unadjusted reimbursement rate for the included orthopaedic foot and ankle procedures increased by 7.3%. During this period, the CPI rose by 52.8%, which is significantly greater than the unadjusted change in foot and ankle procedure reimbursement (*P* < .001) (Table 1). After adjusting for inflation, foot and ankle procedures showed a 29.8% decline in reimbursement between 2000 and 2020. The inflation-adjusted mean Medicare reimbursement amount for physicians across all included foot and ankle CPT codes was $579 in 2000 and $393 in 2020, a difference of $186. During this 20-year period, the annual change for the adjusted mean reimbursement rate for all included foot and ankle procedures was –1.75% per year whereas the average CAGR was –1.88% (Table 2).

**Table 1:** Medicare Reimbursement from 2000 to 2020 for 20 Commonly Performed Foot and Ankle Procedures

CPT Code	Procedure Description	Average Reimbursement in 2000 (in 2000 USD)	Average Reimbursement in 2020 (in 2020 USD)	% Change in Reimbursement 2000-2020 (Unadjusted)
28296	Correction, hallux valgus (bunionectomy), with sesamoidectomy	$668	$545	–18%
28122	Partial excision (craterization, saucerization, sequestrectomy, or diaphysectomy) bone (e.g., osteomyelitis or bossing)	$518	$465	–10%
28299	Correction, hallux valgus (bunionectomy), with sesamoidectomy	$667	$618	–7%
27687	Repair, revision, and/or reconstruction procedures on the leg (tibia and fibula) and ankle joint	$489	$481	–2%
28810	Amputation procedures on the foot and toes	$450	$453	1%
28308	Osteotomy, with or without lengthening, shortening or angular correction, metatarsal	$400	$404	1%
28010	Tenotomy, percutaneous, toe	$218	$221	1%
28124	Partial excision (craterization, saucerization, sequestrectomy, or diaphysectomy) bone (e.g., osteomyelitis or bossing)	$339	$352	4%
28005	Incision procedures on the foot and toes	$584	$612	5%
28313	Repair, revision, and/or reconstruction procedures on the foot and toes	$355	$377	6%
28232	Tenotomy, open, tendon flexor	$244	$256	5%
28272	Capsulotomy	$247	$267	8%
28285	Repair, revision, and/or reconstruction procedures on the foot and toes	$363	$403	11%
28270	Capsulotomy	$317	$355	12%
28001	Incision procedures on the foot and toes	$161	$182	13%
28002	Incision and drainage below fascia, with or without tendon sheath involvement, foot	$295	$339	15%
28750	Arthrodesis, great toe	$540	$618	14%
28234	Repair, revision, and/or reconstruction procedures on the foot and toes	$231	$281	22%
28820	Amputation, toe	$337	$416	23%
28470	Closed treatment of metatarsal fracture	$153	$216	42%
Average		$379	$393	7%

CPT, Current Procedural Terminology; USD, U.S. dollars.

**Table 2:** Medicare Reimbursement From 2000 to 2020 for 20 Commonly Performed Foot and Ankle Procedures

CPT Code	Procedure Description	Average Reimbursement in 2000 (Adjusted for Inflation to Represent 2020 USD)	Average Reimbursement in 2020 USD	CAGR	RSQ	% Change 2000-2020 (Adjusted)
28296	Correction, hallux valgus (bunionectomy), with sesamoidectomy	$1,021	$545	–3.2%	0.84	–47%
28122	Partial excision (craterization, saucerization, sequestrectomy, or diaphysectomy) bone (e.g., osteomyelitis or bossing)	$791	$465	–2.8%	0.83	–41%
28299	Correction, hallux valgus (bunionectomy), with sesamoidectomy	$1,019	$618	–2.6%	0.90	–39%
27687	Repair, revision, and/or reconstruction procedures on the leg (tibia and fibula) and ankle joint	$747	$481	–2.3%	0.75	–36%
28810	Amputation procedures on the foot and toes	$688	$453	–2.2%	0.74	–34%
28308	Osteotomy, with or without lengthening, shortening or angular correction, metatarsal	$611	$404	–2.1%	0.74	–34%
28010	Tenotomy, percutaneous, toe	$334	$221	–2.1%	0.62	–34%
28124	Partial excision (craterization, saucerization, sequestrectomy, or diaphysectomy) bone (e.g., osteomyelitis or bossing)	$518	$352	–2.0%	0.65	–32%
28005	Incision procedures on the foot and toes	$893	$612	–2.0%	0.76	–31%
28313	Repair, revision, and/or reconstruction procedures on the foot and toes	$542	$377	–1.9%	0.67	-–1%
28232	Tenotomy, open, tendon flexor	$372	$256	–1.9%	0.64	–31%
28272	Capsulotomy	$377	$267	–1.8%	0.66	–29%
28285	Repair, revision, and/or reconstruction procedures on the foot and toes	$555	$ 403	–1.7%	0.43	–27%
28270	Capsulotomy	484	$355	–1.6%	0.62	–27%
28001	Incision procedures on the foot and toes	$246	$182	–1.6%	0.68	–26%
28002	Incision and drainage below fascia, with or without tendon sheath involvement, foot	$452	$339	–1.5%	0.86	–25%
28750	Arthrodesis, great toe	$825	$618	–1.5%	0.79	–25%
28234	Repair, revision, and/or reconstruction procedures on the foot and toes	$353	$281	–1.2%	0.58	–20%
28820	Amputation, toe	$515	$416	–1.1%	0.37	–19%
28470	Closed treatment of metatarsal fracture	$233	$ 216	–0.4%	0.32	–7%
Average		$ 579	$ 393	–2%	0.67	–30%

NOTE. Adjusted for inflation using CPI.

CAGR, compound annual growth rate; CPI, Consumer Price Index; CPT, Current Procedural Terminology; RSQ, R squared; USD, U.S. dollars.

None of the CPT codes included in our analysis had a positive average inflation-adjusted reimbursement value. The procedure with the smallest negative decline was 28470 (Closed treatment of metatarsal fracture; –7%). Alternatively, the procedure with the largest decline in reimbursement was 28296 (Correction, hallux valgus-bunionectomy, with sesamoidectomy; –47%). Table 2 shows the average adjusted Medicare reimbursement for all 20 included foot and ankle procedures.

**Fig 1:**
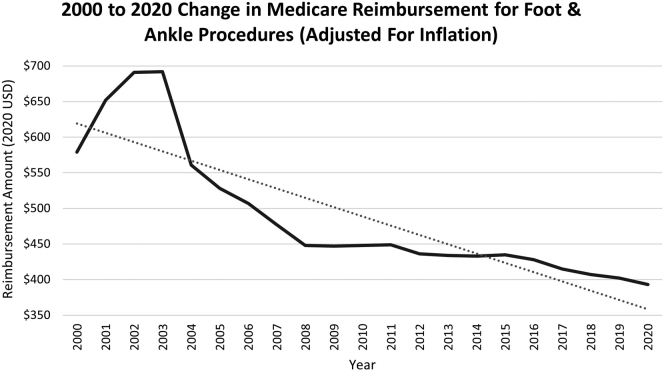
A year-by-year analysis of change in Medicare reimbursement for foot and ankle procedures from 2000 to 2020 after adjusting for inflation.

The average linear R-squared regression value for the included foot and ankle procedures was 0.57, which represents a moderately strong linear and consistent annual decline in Medicare reimbursement rates from 2000 to 2020. The largest single-year decrease in reimbursement across all procedures was between 2003 and 2004. During this year, the average adjusted foot and ankle procedure reimbursement value dropped from $692 to $561 (–19%) (Fig 1). Only 6 of the 20 years included in this analysis noted positive growth in reimbursement amounts, with the highest positive growth from 2000 to 2001 (13%) (Fig 1).

In addition, a sub-analysis demonstrated the average adjusted reimbursement percentage change from 2000 to 2010 and 2010 to 2020. The average inflation-adjusted reimbursement amount across the top 20 foot and ankle procedures decreased by 22% from 2000 to 2010 while decreasing by 12% from 2010 to 2020. The difference between the 2 time periods was statistically significant (*P* < .001) (Table 3).

**Table 3:** Average Change in Reimbursement for Foot and Ankle Procedures From 2000-2010 and 2010-2020

Average % Change in Reimbursement 2000-2010	Average % Change in Reimbursement 2010-2020	*P* Value
–22.2%	–11.5%	<.0001

## Discussion

We found that Medicare reimbursement rates for the 20 most commonly performed foot and ankle procedures have decreased by nearly 30% from 2000 to 2020 when adjusting for inflation, with the average adjusted reimbursement for all included procedures decreasing from $579 to $393. In addition, only 6 of the 20 years studied demonstrated positive reimbursement growth. The 2 procedures with the largest decreases in reimbursement were “bunionectomy with sesamoidectomy” and “partial excision of bone.” These decreases in Medicare reimbursement are further contextualized by reports of increasing practice expenses, salaries, and the substantial cost of integrating new technologies in medical practices.^16^

Medicare is the largest payer in our current health care system, and recent estimates have shown Medicare is responsible for almost 30% of total revenue in hospitals.^12^ Changes in health care policy and decreased reimbursement by Medicare may result in hospitals adopting cost-reduction measures and also could impact how physicians practice and the quality of care they provide. As evidence of these adjustments, a survey done in 2018 by The Physicians Foundation found 17% of specialists do not see and/or limit Medicare patients in their practice.^17^ Further, changes in Medicare reimbursement to physicians can have a profound ripple effect on the health care system, as Medicare often influences reimbursement rates by other payers.^18^ The alarming decrease in reimbursement to orthopaedic foot and ankle physicians, coupled with increases in practice expenses, warrants further attention to ensure continued access to quality health care services for patients.

The average 22% decrease in reimbursement seen in our study from 2000 to 2010 is likely attributable to the Sustainable Growth Rate enacted under the Balanced Budget Act of 1997, which operated on a fee-for-service (FFS) model of reimbursement and significantly decreased Medicare spending and physician reimbursement by Medicare.^19^ Additionally, the largest decrease in our study (19% in 1 year) was seen from 2003 to 2004 and could be explained by additional legislation enacted in 2002 under Sustainable Growth Rate to significantly cut Medicare spending. Following the enactment of the Balanced Budget Act of 1997, health care experts began to question the FFS model of reimbursement, which may have incentivized physicians to overuse services with less attention paid to the necessity and quality of the services.^20^

In 2015, SGA was replaced by the Medicare Access and Children’s Health Insurance Program Reauthorization Act of 2015 (MACRA), replacing FFS with value-based care. MACRA provided flexible reimbursement models for physicians and initiated a 0.5% increase in the conversion factor to increase Medicare reimbursements each year.^21^ In our study, 2010 to 2020 was associated with a smaller decrease of 12% in reimbursement to physicians and the smaller decrease in reimbursement could be due to transitioning to MACRA.

MACRA gave eligible providers 2 different options for reimbursement: the alternative payment model or the merit-based incentive payment system.^22^ The merit-based incentive payment system and alternative payment model programs both provide additional compensation for providing greater quality care with an improved focus on patient outcomes. However, the efficacy of these programs in achieving these goals warrants further research. A recent study by Shih et al.^23^ concluded the pay-for-performance model did not improve surgical outcomes in the hospitals studied. Risk prediction is also an area of debate, as a study by Edelstein et al.^24^ showed current risk stratification tools poorly predict surgical complications.

With the exploration of new payment models in healthcare, an understanding of physician reimbursement trends is essential for continued progress toward a sustainable health care model with accurate risk prediction, agreeable quality measures, and excellent patient outcomes.^25^ The findings in our study are similar to findings reported in other specialties. For example, Haglin et al.^6^ reported a 25% decrease in physician reimbursement for neurosurgical procedures from 2000 to 2018. In addition, Mayfield et al.^26^ reported a 40% decrease from 2000 to 2020 for primary total knee arthroplasty and 37% for hip arthroplasty. However, other fields have reported increases, such as radiation oncology, with an increase of 14% in reimbursement from 2012 to 2015.^4^

These changes in Medicare reimbursement among specialties are largely due to inflation, health care policy, a stagnant conversion factor, and adjustments made by the Specialty Society Relative Value Update Committee.^8^^,^^27^, ^28^, ^29^ The Relative Value Update Committee is a board of 32 volunteer multispecialty physicians who advise CMS on how to value medical services.^28^ Following these suggestions, CMS ultimately decides on medical service reimbursement adjustments. Further study of the financial trends seen in our study are needed as reimbursement models and the field of orthopaedic surgery continue to evolve. This study can help contextualize Medicare reimbursement changes in foot and ankle surgery compared to other subspecialties in orthopaedic surgery.

### Limitations

This study has limitations. One is the use of the Medicare database, which is a single database from a single payer. This study only analyzed the 20 most commonly performed foot and ankle procedures. Geographic analysis was not possible and warrants future study.

## Conclusions

From 2000 to 2020, inflation-adjusted Medicare reimbursement for foot and ankle surgery decreased by 30%.

## Supplementary Data

ICMJE author disclosure forms
